# Excellence in dental research: nominated scholars for the Nobel Prize 1901-1950 with a focus on Lady May Mellanby (1882-1978) and Walter Hess (1885-1980)

**DOI:** 10.1038/s41415-022-3996-1

**Published:** 2022-06-10

**Authors:** Lena Hense, Alfons Hugger, Nils Hansson

**Affiliations:** 4141598015001 Department for the History, Philosophy, and Ethics of Medicine, Faculty of Medicine, Heinrich-Heine University Duesseldorf, Moorenstr 5, 40225 Duesseldorf, Germany; 4141598015002 Department of Prosthodontics, Düsseldorf University Hospital, Heinrich-Heine University Duesseldorf, Moorenstr 5, 40225 Duesseldorf, Germany

## Abstract

Why has no dentist received the Nobel Prize so far? To answer that question, we need to take a closer look at the prize candidates. This article presents an overview of scholars in the field of dental research who were nominated for the Nobel Prize in Physiology or Medicine during the first half of the twentieth century. Drawing on archival sources in the archive of the Nobel Committee, we focus on the physiologist, Lady May Mellanby (1882-1978) and the dentist, Walter Hess (1885-1980). While Hess did not reach the shortlist, Mellanby was judged 'prize-worthy' by the Nobel Committee for Physiology or Medicine but she never received the award in the end. In this paper, we discuss the impact of their work among dentists.

## Key points

- Recent historical research argues that nominees, not only the prize-winners of renowned awards, can help to reconstruct trends in medicine over time.
- This paper takes a new approach on the history of dental research and shows why the oral health researchers Lady May Mellanby and Walter Hess were runners-up for the Nobel Prize.
- On a more general level, the article raises questions about the gender award gap and why so few women have received the most prestigious prizes in medicine.

## Introduction

There are several prestigious awards in dentistry, such as the Distinguished Scientist Award which is awarded by the International Association of Dental Research, occasionally paraphrased as the 'Nobel Prize of dentistry',^1^^,^^2^^,^^3^^,^^4^ linking it to the most renowned scientific award worldwide.^5^ Since 1901, when the first Nobel Prize was awarded, 222 laureates (1901-2020) have received the Nobel Prize in Physiology or Medicine for discoveries of 'the greatest benefit to humankind'; the key phrase in Alfred Nobel's will. Among the laureates, physicians and scientists from all kinds of medical fields are listed, from basic to clinical research, but so far there has been no dentist. Why? Our research group has analysed nominations and Nobel Committee evaluations within this prize category in different specialities, such as cardiovascular research,^6^ surgery,^7^ neurology and pharmacology.^8^^,^^9^ Very few scientific articles deal with dentistry and the Nobel Prize. A recent article analysed the life and work of the German dentist and physician Carl Röse (1864-1947), the first known dentist nominated for the Nobel Prize.^10^ Röse was nominated for the first and last time in 1907 for research on cariology but he was not of particular interest to the Nobel Committee.

This article presents an overview of scholars in the field of dental research who were nominated for the Nobel Prize in Physiology or Medicine during the first half of the twentieth century, with a particular focus on the British researcher Lady May Mellanby (1882-1978). She is of special interest not only because of her research, which, as we shall see, was judged prizeworthy by the committee, but also because of her position as a female scientist during the first decades of the twentieth century.

## Methods

For this article, we used the Nobel nomination database and searched the category 'physiology or medicine' from 1901-1950 to pinpoint scholars (Nobel nominees and nominators) linked to dental research.^11^ We then collected nomination letters in the archive of the Nobel Committee for Physiology or Medicine in Stockholm to explore nomination reasons and to reconstruct what was considered to be excellent dental research in the context of the Nobel Prize. The article also draws on Mellanby's publications and secondary literature.

## Results

We identified three dental researchers who were nominated for the Nobel Prize in physiology or medicine during the first half of the twentieth century: The Swiss dentist, Walter Hess (1885-1980) (primarily because of his work on preservation of teeth, especially the histology of the root canal), the aforementioned German dentist, Carl Röse (1864-1947) and Lady May Mellanby (1882-1978). In the following, we will reconstruct the candidacies of Hess and Mellanby.

## Walter Hess

In 1885, Walter Hess was born in Amriswil, Switzerland. Educated as a dental technician, Hess decided to become a dentist and was enrolled at the University of Zurich in 1904. He passed the official exam for dental students in 1908. After receiving his state licence to practise dentistry, Hess worked as an assistant doctor at the dental institute until the following year.^12^ From 1909-1911, Walter Hess studied medicine in Zurich and finished his dissertation thesis one year later. In the following years, Hess went abroad for further dental training in Marseille and London. After coming back to Zurich in 1913, Hess opened his own dental office and worked as a lecturer and researcher at the Dental Medicine Institute at the University of Zurich.^12^ He completed his habilitation thesis in 1917 (in conservative dentistry) and became director of the institute in 1931, a position he held until 1953.^12^^,^^13^ His influence in the scientific community was demonstrated in other gate-keeping positions and honours. Hess was the editor-in-chief of the *Swiss Dental Journal* for more than 40 years, from 1921-1962.^12^ Walter Hess was appointed to an honourary member of several dental societies, such as the British Dental Association, the Tokyo Dental College and the German Association for Dental, Oral and Maxillofacial Medicine. He died in 1980, aged 95.

### The Nobel Prize nomination for Walter Hess

Hess was nominated for the Nobel Prize in 1949 by the Swiss dentist and Zurich colleague, Alfred Gysi (1865-1957). Gysi nominated Hess for his work on preservation of teeth and especially the histology of the root canal. Furthermore, Hess was nominated for his research on the effects of devitalisation agents, antiseptics and chemotherapeutic agents on the pulp and periapical tissue. These research interests had been a long-time focus of Hess. In 1917, Hess defended his habilitation thesis about his further investigations on the anatomy of the root canal, including the analysis of the fine ramifications at the foramen apicale.^14^ His focus was to observe the impact of the age and development to the anatomic form and number of the root canals. Of particular interest is that his observations contained extensive information about every tooth type (incisor, canine, premolar, molar). In the following years, Walter Hess focused his clinical research on mortal pulp amputation and pulpectomy.^15^ In the early 1930s, Hess turned away from this topic and concentrated on the maintaining of the vitality of the pulp and questioned its regenerative capacity.^16^^,^^17^^,^^18^ His research focus then became pulpotomy and iontophoresis.^19^

His early publications about the anatomy and development of the human root canal, which were of interest to the Nobel Prize Committee, are to this day cited and are still regularly mentioned in dental journals and endodontology textbooks.^12^^,^^20^^,^^21^^,^^22^^,^^23^ Nevertheless, the members of the Committee, Gösta Häggqvist (anatomist and histologist) and Göran Liljestrand (pharmacologist), did not judge him prizeworthy. Häggqvist wrote in his statement that researchers like Carabelli (1884), Mühlreiter (1891) and Preiswerk (1901) already studied successfully in this field and that Hess' work was not, in a Nobel context, truly pioneering.^24^ Liljestrand wrote that the nominator added around 70 publications of which some were written by Hess' employees and not all by himself. He also noted that the nomination of Walter Hess did not present one great discovery but rather several single minor discoveries and research results.^25^ Thus, both jury members viewed his work as a valuable contribution to clinical dental research but not original enough for a Nobel Prize. In the same year, another Walter Hess (1881-1973), the Zurich physiologist, received the Nobel Prize in Physiology or Medicine for his discovery of the functional organisation of the interbrain.^26^

## Lady May Mellanby

In 1882, May Tweedy was born in London. She spent part of her childhood in Russia due to her father's involvement in the oil industry.^27^ After moving back to London, she attended the London schools Hampstead and Bromley High School,^27^^,^^28^ both schools exclusively for girls. In 1902, she went to Girton College (University of Cambridge) and attended both parts of the natural science Tripos in 1905 and 1906. She received the equivalent second-class degree.^27^ Although some women in the UK were allowed to vote in political elections from 1928 onwards, they were still not allowed to receive their degrees from the University of Cambridge until 1948.^29^

Subsequently, May Tweedy worked as a research fellow and later as a lecturer at Bedford College for women at the University of London.^27^^,^^28^ At Bedford College, she carried out studies on gastric secretion together with John S. Edkins (1863-1940), later renowned as the discoverer of the peptide hormone gastrin.^30^ May Tweedy continued her work as a lecturer at Bedford until her marriage with the London physiologist, Edward Mellanby in 1914. After the marriage, they both moved to Sheffield. Since the First World War, May Mellanby worked under the auspices of the Medical Research Committee at the Brown Animal Sanatory Institution and then in Sheffield from 1920-1933.^27^ It was here when her husband completed his work relating vitamin D to the disease of rickets.^31^ From 1935-1948, Edward Mellanby acted as full-time secretary of the Medical Research Council (MRC). During this time, their own research was continued at the weekends at the Mill Hill Nutrition Laboratory.^32^ In 1937, May Mellanby became a Lady when Edward Mellanby was knighted. Lady May Mellanby died on March 5, 1978.^27^^,^^28^

### Research interests

In addition to the nutritional studies with her husband, May Mellanby received Medical Research Council funding for research on nutritional influences on dental development. In 1918, May Mellanby published research results showing that dietetic food factors control the structure of teeth in puppies.^33^ After Mellanby had shown in her animal experiments that the development of the structure of teeth is controlled by diet and by environmental factors, she showed that food substances containing vitamin D (at first mainly described as antirachitic or calcifying vitamin) stimulated the calcification of teeth.^34^^,^^35^ In 1928, Lady May Mellanby published her further research showing that the addition of vitamin D had a significant influence in preventing the initiation of a new carious lesion, limiting the spread of disease and arresting the carious process in children.^35^ Her studies argued that the presence and structure of secondary dentine indicates a good defensive force and that the quality of formed secondary dentine is also related to diet.^36^

From 1944-1962, Mellanby did observations of five-year-old children with regards to dental structure and the resistance to dental disease. She compared children from private schools, county council schools and residential homes/day schools. Furthermore, she compared the differences of her observations before (1929), during (1943) and after the Second World War (1945*),*^37^^,^^38^ taking both different social classes and ethnic groups into account.

Her research articles were summarised in the chapter 'Dental structure and disease' of the book *Nutrition and disease* (1934), written by Edward Mellanby.^39^ The research that led to her Nobel Prize nominations was published in three MRC special reports in the years 1929, 1930 and 1934.^34^^,^^40^^,^^41^ From 1918-1962, Mellanby published more than 20 scientific articles in renowned journals, such as the *British Medical Journal* and *The Lancet* (first author in 19 of them).^33^^,^^35^^,^^36^^,^^37^^,^^38^^,^^42^^,^^43^^,^^44^^,^^45^ According to the Web of Science, her articles have to date been cited 281 times. Of special interest here are her publications between 1918-1932, which led her to the nomination for the Nobel Prize (Table 1). Although her relatively low citation level (Edward Mellanby's publications were cited almost 3,000 times according to Web of Science), she had a lasting impact on the scientific community through her special reports for the MRC, which reported all her experimental research results on the topics from above.

**Table 1:** Mellanby's most cited articles (authored before 1939) (published before the Nobel nomination in 1939)

Number	Year	Number of citations	Author	Name of publication	Journal name	Position in journal
1	1918	36	M. Mellanby	'The influence of diet on teeth formation'	*The Lancet*	Volume 2; pages 767-770
2	1924	16	M. Mellanby, C. L. Pattison, J. W. Proud	'The effect of diet on the development and extension of caries in the teeth of children'	*British Medical Journal*	Volume 1,924; pages 354-355
3	1928	49	M Mellanby, C. L. Pattison	'The action of vitamin D in preventing the spread and promoting the arrest of caries in children'	*British Medical Journal*	Volume 1,928; issue 2; pages 1079-1082
4	1928	45	M. Mellanby	'The influence of diet on the structure of teeth'	*Physiological Reviews*	Volume 8; issue 4; pages 545-577
5	1932	16	M. Mellanby, C. L. Pattison	'Remarks on the influence of a cereal-free diet, rich in vitamin D and calcium on dental caries'	*British Medical Journal*	Volume 1,932; issue 1; pages 507-510
Note: List obtained by access to Web of Science on 20 October 2020						

### Nobel Prize nominations for Lady May Mellanby

In 1939, May and Edward Mellanby were jointly nominated for the Nobel Prize in Physiology or Medicine for their work on the relation of dietary deficiencies to human diseases: May Mellanby especially for her research on dental structure and dental disease. While May Mellanby was nominated three times in 1939, Edward Mellanby was nominated four times in 1939 and five times in 1947 for his work on rickets (including his work on the antirachitic vitamin) and the nervous condition produced by a lack of vitamin A.

In 1939, Göran Liljestrand, secretary of the Nobel Committee, signed a 'special investigation' he had written to determine whether - in his view - Edward and Lady May Mellanby's work on avitaminosis should be deemed prizeworthy. In 23 type-written pages, he discussed their publications in renowned journals, as well as more comprehensive special reports on calcium-phosphorus-balance and rickets in dogs. Liljestrand emphasised that May Mellanby's studies have been of great importance for humanity.^46^ He also assumed that she had provided results of basic research that most probably would lead to practical solutions to prevent and control caries. Furthermore, he wrote 'although some of the studies were published as early as in 1918, it has only in recent years become apparent that they are relevant in practice, which is a prerequisite for prizeworthyness. To this I can add a statement, which in my view without a doubt is correct: it is also not unreasonable to claim that her work has not only revolutionised dental science but that she has continued to lead the field in discovery throughout the world for many years in this special subject'.

Thus, Liljestrand concluded that Lady May Mellanby's discoveries of the importance of vitamin D and A for the development of teeth and for preventing teeth (disease) were worthy of a Nobel Prize in Physiology or Medicine. He then suggested that Edward and May Mellanby should share a Nobel Prize.^47^ In the end, however, the jury agreed on the German pathologist, Gerhard Domagk, who received the Prize in 1939 for the discovery of the antibacterial effects of prontosil (sulphonamides).^48^

By evaluating those candidates, who were of particular interest for the members of the Nobel Committee in Physiology or Medicine in 1939, it is noticeable that Mellanby was the only woman among the 'shortlisted candidates' (Table 2). During the first half of the twentieth century, women were highly under-represented in higher professional (medical) positions, a condition that remains to this day. Men were mainly nominated for the Nobel Prize or have been nominators for the Prize.^49^ To this day, only 12 women out of 222 laureates have received the Prize and only one female scientist, the biochemist Gerty Cori, received the Nobel medal (physiology or medicine) in the first half of the twentieth century.

**Table 2:** Shortlisted candidates for the Nobel Prize in the category 'physiology or medicine' 1939

Name	Lifetime	Nationality	Profession	Motive of nomination
Frederick C. Bawden	1908-1972	British	Plant biology, virology	Work on production of plant disease generating virus in crystallised form
Norman W. Pirie	1907-1997	British	Biochemistry	Work on production of plant disease generating virus in crystallised form
Gerhard Domagk	1895-1964	German	Pathology	For the discovery of the antibacterial effects of prontosil (got the prize 1939)
Gösta Forssell	1876-1950	Swedish	Radiology	Work on the mucosal patterns of the stomach and intestines
Evarts A. Graham	1883-1957	American	Surgery	Discovery of visualisation of the gallbladder, treatment of empyema, tuberculosis, bronchiectasis, carcinoma of the lung and adhesive pericarditis
Walter Rudolf Hess	1881-1973	Swiss	Physiology	Work on the physiology of the brain stem, the regulation of sleep, respiration and circulation; work on the coordimeter method
Corneille J. Heymans	1892-1968	Belgian	Pharmacology	Work on the regulation of respiration and circulation
Edward Mellanby	1884-1955	British	Physiology	Work on the relation of dietary deficiencies to human diseases, rickets and the nervous condition produced by lack of vitamin A
May Mellanby	1882-1978	British	Physiology	Work on the relation of dietary deficiencies to human diseases; dental structure and dental disease
Hermann J. Muller	1890-1967	American	Molecular biology	Production of mutations by means of x-ray irradiation
John H. Northrop	1891-1987	American	Biochemistry	The purification, crystallisation and method of action of pepsin and trypsin; purification of their pro-enzymes
Alfred N. Richards	1876-1966	American	Pharmacology	Work on the physiology of the kidneys (mechanism of renal secretation)
Thomas M. Rivers	1888-1962	American	Virology	Work on virus (virus III infections in rabbits, in vitro cultivation of viruses and vaccination against psittacosis)
Peyton Rous	1879-1970	American	Pathology	Work on the filterability of the existing agent of certain fowl tumours and the carcinogenic effect of papilloma virus injected in rabbits
Wendell M. Stanley	1904-1971	American	Chemistry, biochemistry	The purification and crystallisation of viruses
James B. Sumner	1887-1955	American	Biochemistry	The purification and crystallisation of the enzyme urease and the study of its action
Hugo A. Theorell	1903-1982	Swedish	Biochemistry	Work on the yellow ferment, especially its resynthesis

### The commemoration and legacy of May Mellanby

Although May Mellanby never became a Nobel laureate, her work was recognised in many ways. She received an honourary Doctor of Science degree by the universities of Sheffield (1933) and Liverpool (1934) as well as a Cambridge Doctor of Science. In 1935, she and her husband were awarded the Charles Mickle fellowship of Toronto University.^27^^,^^28^^,^^50^ She was elected to the Physiological Society in 1956 and became an honourary fellow at Girton in 1958.^27^^,^^28^^,^^31^ As her final honour, she received the Science Award of the International Association for Dental Research in 1975.^27^^,^^28^

*The Times* (1978) stated in an obituary that Mellanby 'made a substantial personal contribution to research on diet and teeth'.^31^ Her research appears in books dealing with these topics, such as *International women in science: a biographical dictionary to 1950*; *A survey of the literature of dental caries;* and *Women physiologists*.^27^^,^^28^^,^^51^

Also, a more recent scholarship re-examined Mellanby's historical microscope slides and described her work as 'pioneering'.^52^ A further systematic review and meta-analysis^53^ which was published in 2013 examined the impact of vitamin D on dental caries prevention and included Mellanby's publications from 1924 and 1926.^36^^,^^54^ The author Phillippe P. Hujoel concludes, like Mellanby, that vitamin D in childhood may reduce the incidence of caries.

That said, her research has also been marked by controversies. Until the early twenty-first century, there was an obvious disagreement between clinicians and basic scientists regarding the interpretation of her research: clinicians argued that experimental results could not be applied to the understanding or treatment of human diseases in general^55^ and May Mellanby was criticised, in particular, for the application of the vitamin theory to the development and arrest of dental caries.^41^^,^^55^^,^^56^

## Why hasn't a dentist received the Nobel Prize so far?

This article demonstrates that only few dental researchers were nominated for the Nobel Prize during the first half of the twentieth century and none of them received it. Why? One reason is the pool of nominators, which only included two dental researchers in the examined time period. Besides Alfred Gysi (mentioned above), the German dentist Max Apffelstaedt (1863-1950) proposed the bacteriologist and immunologist Paul Uhlenhuth (1870-1957) in 1929, with no reference to dental research. Another reason might also be that, so far, no dentist has been part of the Nobel Prize Committee. Further research aims at analysing Nobel Prize nominations for and by dental researchers during the second half of the twentieth century to reconstruct possible further candidates in the field.
